# Hepatocellular carcinoma with vertebral metastasis in a child with hepatitis B virus infection: a case report

**DOI:** 10.1186/s13256-019-2099-3

**Published:** 2019-05-27

**Authors:** Tinsae Alemayehu, Daniel Hailu

**Affiliations:** Department of Pediatrics and child health, College of health sciences, Addis Ababa University, Addis Ababa, Ethiopia

**Keywords:** Hepatocellular carcinoma, Hepatitis B, Metastasis, Child, Ethiopia

## Abstract

**Background:**

Hepatocellular carcinoma in children with hepatitis B virus infection is rarely reported. Metastases to the vertebrae are an even more unusual phenomenon.

**Case presentation:**

We report on a case of a 10-year-old Ethiopian boy with hepatitis B infection presenting with paraplegia and incontinence of 10 days’ duration. A diagnosis of hepatocellular carcinoma with vertebral metastases was confirmed with serum α-fetoprotein, fine-needle aspirate cytology, and abdominal imaging.

**Conclusion:**

Surveillance of children not immunized against hepatitis B virus prevents infection and its complications, such as hepatocellular carcinoma. Among children in endemic countries prone to development of hepatocellular carcinoma, metastatic disease can present as sudden weakness of extremities with radiologic findings of vertebral body collapse.

## Background

Hepatocellular carcinoma (HCC) is one of the top five most common cancers worldwide. A high incidence is seen in sub-Saharan Africa and South-East Asia. Among adults, male sex, advanced age, alcohol use, hemochromatosis, and aflatoxin exposure are potential risk factors. Superseding all risk factors, at any age, are chronic hepatitis B and C infection [1].

Childhood hepatitis B infection is mostly acquired via the perinatal route. Sequelae of chronic infections such as cirrhosis and HCC have rarely been reported in children, with those few reports being notably of adolescents with genotype B infection from South-East Asia. Metastatic HCC occurs through direct, hematogenous, or lymphatic spread. Frequent sites of dissemination are lungs or lymph nodes. Vertebral metastases are infrequently reported [2]. We report a case of a 10-year-old boy with hepatitis B infection who had HCC and vertebral metastases.

## Case presentation

A 10-year-old Ethiopian boy presented to Tikur Anbessa Specialized Hospital, Addis Ababa, Ethiopia, with a sudden onset of weakness over his lower extremities. It had started 10 days earlier, accompanied by incontinence of urine and feces. He also had long-standing epigastric pain. He had no fever, diarrhea, cough, unconsciousness, abnormal body movements, or trauma. He had no close contact with a chronic cougher. He had received all scheduled vaccines during infancy, including four doses of oral polio vaccine, except hepatitis B vaccine, because he was born 1 year earlier than the incorporation of a routine three-dose series of hepatitis B vaccines into the national vaccination schedule. (He presented to our center in 2016.) Upon examination, he had an axillary temperature of 38.0 °C and tachycardia (115 beats per minute). He had a hard and tender hepatomegaly of 16-cm total span (10 cm below the right costal margin). Neurologic examination revealed a sensory level at T10, power of 0/5 of bilateral lower extremities, areflexia, and hypotonic anal tone.

Investigations confirmed a normal complete blood count and erythrocyte sedimentation rate of 25 mm/hr. The patient’s liver enzymes were elevated: alanine aminotransferase 160 U/L and aspartate aminotransferase 136 U/L. Alkaline phosphatase was 761 U/L, and serum albumin was 3.4 mg/dl. His coagulation profile, renal function, serum electrolytes, blood and urine cultures, and human immunodeficiency virus and hepatitis C serologies were negative. His hepatitis B surface antigen was positive. Additional serologic testing to identify the state of his hepatitis B infection was not accessible.

Thoracolumbar magnetic resonance imaging outlined a T9 vertebral body collapse with marrow signal change showing T1 isointensity and T2 heterogeneous hyperintensity. An epidural and paravertebral soft tissue swelling extending from T7 to T11 with postcontrast enhancement was seen to significantly compress the spinal cord. Differential diagnoses of tuberculosis spondylitis and metastases were considered.

**Fig. 1:**
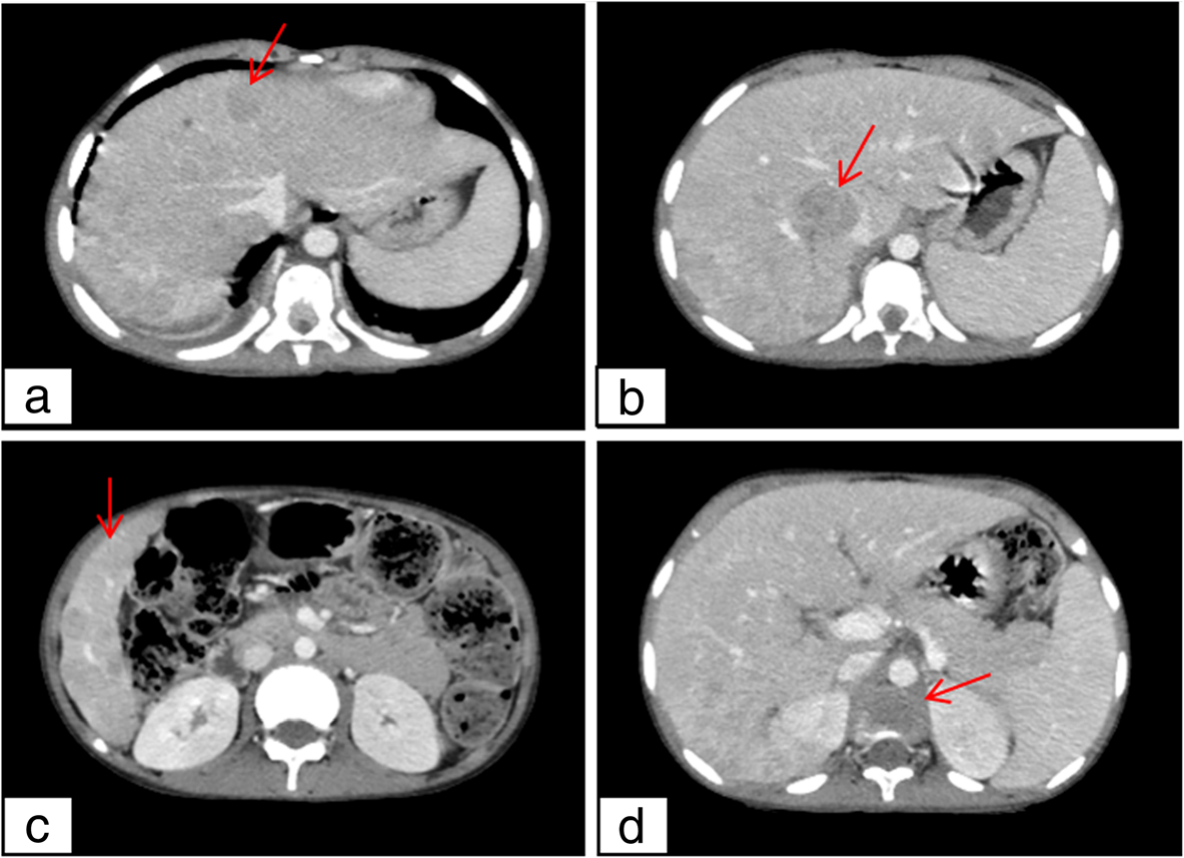
**a**, **b** and **c**, the arrows point to “multiple solid hepatic masses”. **d** the arrow points to “the vertebral body collapse”

Workup for a primary malignancy showed elevated serum lactate dehydrogenase (599 U/L) but normal serum uric acid, chest x-ray, and abdominal ultrasound. Computed tomography of the abdomen (Fig. 1a–d) revealed multiple well-defined, different-sized, solid hepatic masses with invasion and thrombosis of the right portal vein branch. The liver had a heterogeneous attenuation. The thoracic vertebral body had collapsed with an adjacent soft tissue mass extending into the spinal canal and prevertebral space.

Serum α-fetoprotein (AFP) was greater than 40,000 IU/L, and an ultrasound-guided fine needle aspirate showed moderately pleomorphic polygonal cells with round nuclei and ample granular eosinophilic cytoplasm. Thus, a diagnosis of HCC with vertebral metastases was confirmed. The boy’s parents were counseled on the prognosis of the illness, following which they decided against further medical care and opted for home palliative care.

## Discussion and conclusions

HCC is a rare complication of chronic hepatitis B infection in children. None of an Italian cohort of 108 children with hepatitis B infection aged 2–18 years who were observed for up to 24 years developed HCC. Affected children present with localized pain, nausea, vomiting, weight loss, and jaundice. Children diagnosed at an older age have a poor prognosis. Median age at diagnosis is 12 years [3].

Elevated liver enzymes are seen in 40% of children with HCC. Raised serum AFP (higher sensitivity when more than 20 ng/ml) is seen in more than half of patients. Imaging and histology are the main diagnostic tools. Large pleomorphic tumor cells and tumor giant cells with different levels of differentiation are seen in histologic examination. Nervous system involvement in metastatic HCC is reported in 0.28% of cases. Of these, one-third involve the spine, making it an extremely rare observation. The thoracic and lumbar spine are commonly involved with collapse of vertebral bodies and extension into the spinal canal and severe cord compression. T1-weighted images show low-signal lesions [4].

Complete surgical resection should be attempted. Chemotherapy with cisplatin and doxorubicin may be given following complete resection of small tumors (stages 1 or 2). If nonresectable, chemotherapy followed by resection with or without liver transplant may be offered. Chemotherapy has proved futile for stage 4 (metastatic) disease. Overall prognosis is good for stages 1 and 2 disease (5-year survival of 90% and a 2-log reduction of serum AFP following chemotherapy even when lesions are unresectable). Metastatic disease carries a poor prognosis (5-year survival of 20%). Early laminectomy prevents complete paralysis. Radiotherapy is recommended for vertebral metastases [5].

Ultrasound surveillance for HCC every 6 months is indicated for hepatitis B surface antigen carrier children, especially those with cirrhosis or a family history of HCC. Our patient had not been vaccinated against hepatitis B. There was a delay in presentation for care for the metastatic HCC, hence aggravating his prognosis. Further serologies could not be tested to identify the stage of the child’s hepatitis B infection (anti-hepatitis B core antibody, hepatitis B antigen E [HBe] antigen, anti-HBe antibody, and hepatitis B viral load).

In Ethiopia, screening of children born prior to 2007 and immunizing if a negative result is obtained effectively prevent acquisition of hepatitis B infection and its complications, such as HCC. Metastatic HCC should be considered in high-risk children residing in countries endemic for hepatitis B carriage, such as Ethiopia, and who have sudden onset of weakness of the extremities and imaging findings of vertebral body collapse.

## Abbreviations

AFP: α-Fetoprotein
Anti-HBe: Anti-hepatitis B antigen E
HCC: Hepatocellular carcinoma
